# Long-term follow-up to assess criteria for ovarian tissue cryopreservation for fertility preservation in young women and girls with cancer

**DOI:** 10.1093/humrep/dead060

**Published:** 2023-04-03

**Authors:** Kathleen Duffin, Ruth Howie, Tom W Kelsey, Hamish B Wallace, Richard A Anderson

**Affiliations:** Biomedical Sciences, University of Edinburgh, Edinburgh, UK; Edinburgh Fertility Centre, Simpsons Centre for Reproductive Health, Royal Infirmary of Edinburgh, Edinburgh, UK; Department of Computer Science, University of St Andrews, St Andrews, UK; Department of Child Life and Health, University of Edinburgh, Edinburgh, UK; MRC Centre for Reproductive Health, Queens Medical Research Institute, University of Edinburgh, Edinburgh, UK

**Keywords:** fertility preservation, childhood cancer, premature ovarian insufficiency, ovarian tissue cryopreservation, cancer survivorship

## Abstract

**STUDY QUESTION:**

Do the Edinburgh Selection Criteria correctly identify female cancer patients under the age of 18 who are at risk of premature ovarian insufficiency (POI) as candidates for ovarian tissue cryopreservation (OTC)?

**SUMMARY ANSWER:**

Patient assessment using these criteria accurately identifies those at risk of POI, who can be offered OTC and future transplantation as a means of fertility preservation.

**WHAT IS KNOWN ALREADY:**

Treatment for childhood cancer can have adverse consequences on future fertility; at the time of diagnosis, fertility risk assessment should be undertaken in order to identify patients to whom fertility preservation should be offered. The Edinburgh selection criteria, based on planned cancer treatment and patient health status, are utilized to identify those at high risk and therefore eligible for OTC. However, this procedure is not without risk and there are few data on the efficacy of the procedure in prepubertal patients. As such, long-term follow-up of reproductive outcomes is necessary, to ensure that OTC is being offered appropriately.

**STUDY DESIGN, SIZE, DURATION:**

Cohort study encompassing all females diagnosed with cancer under the age of 18 in South East Scotland, from 1 January 1996 to 30 April 2020. Patients were followed up for reproductive outcomes to assess for diagnosis of POI.

**PARTICIPANTS/MATERIALS, SETTING, METHODS:**

A total of 638 eligible patients were identified; patients under the age of 12 or deceased before the age of 12 were excluded from the study, leaving a study population of 431 patients. Electronic records were reviewed for reproductive function, assessed by current menstruation, pregnancy (in the absence of POI diagnosis), reproductive hormone measurements, pubertal progression, or diagnosis of POI. Patients on hormonal contraception (other than for treatment of POI or panhypopituitarism with no history of gonadatoxic treatment) were excluded from analysis (n = 9). Analysis on remaining 422 patients was carried out using the Kaplan–Meier methods, with POI as the defined event, and Cox proportional hazards model.

**MAIN RESULTS AND THE ROLE OF CHANCE:**

In the study population of 431 patients, median ages at diagnosis and analysis were 9.8 and 22.2 years, respectively. Reproductive outcomes were unavailable in 142 patients; the assumption was made that these patients did not have POI, but a subanalysis excluding these patients was also performed. Of the 422 patients aged >12 at analysis and not taking hormonal contraception, OTC was offered to 37 patients and successfully performed in 25 patients. Of the 37 patients offered OTC (one at time of relapse), nine (24.3%) developed POI. Of the 386 not offered OTC, 11 (2.9%) developed POI. The probability of developing POI was significantly higher in those offered OTC (hazard ratio [HR] 8.7 [95% CI 3.6–21]; *P* < 0.0001), even when those patients with unknown outcomes were excluded from the analysis (HR 8.1 [95% CI 3.4–20]; *P* < 0.001). All patients offered OTC who developed POI did so after treatment for primary disease; in those not offered OTC, five patients (45.5%) developed POI after treatment for disease relapse.

**LIMITATIONS, REASONS FOR CAUTION:**

A significant number of patients had unknown reproductive outcomes; many of these patients were engaged in ongoing follow-up but did not have documented reproductive assessment. This may have introduced bias to the analysis and highlights the need for reproductive follow-up as part of routine cancer aftercare. In addition, the relatively young age of the patient population and short duration of follow-up in some cases demonstrates the need for ongoing follow-up of this cohort.

**WIDER IMPLICATIONS OF THE FINDINGS:**

The prevalence of POI after childhood cancer is low, but the Edinburgh selection criteria remain a robust tool for selecting those at high risk at the time of diagnosis, to offer OTC appropriately. However, disease relapse necessitating more intensive treatments remains a challenge. This study additionally highlights the importance of routine assessment and documentation of reproductive status in haematology/oncology follow-up.

**STUDY FUNDING/COMPETING INTEREST(S):**

K.D. is supported by a CRUK grant (C157/A25193). This work was undertaken in part in the MRC Centre for Reproductive Health, (supported by MRC grant MR/N022556/1). R.A.A. has received consulting fees from Ferring and Roche Diagnostics; payment from Merck and IBSA for educational events; and laboratory materials from Roche Diagnostics. The other authors have no competing interests to declare.

**TRIAL REGISTRATION NUMBER:**

N/A.

## Introduction

Childhood malignancy is rare but increasing, with around 1900 new cases diagnosed each year in the UK. However, treatments continue to advance, resulting in improved survival rates, with over 75% of patients disease free at 10 years (Children with Cancer UK, 2021). These improved survival rates are associated with an increasing number of young people facing a wide variety of long-term complications, or late effects, from their treatment. One of these late effects is the impact on future reproductive function and fertility, which is a key concern of cancer survivors (Young *et al.*, 2019).

The risk of gonadotoxicity following some treatments for cancer is well recognized. However, the overall chance of future parenthood in survivors of childhood cancer remains high. Data from the US Childhood Cancer Survivor Study (CCSS) show that female cancer survivors not exposed to radiotherapy have similar rates of pregnancy and livebirth to their siblings, except when exposed to busulfan, high dose lomustine or high cumulative doses of other alkylating agents (Chow *et al.*, 2016). The effects of radiotherapy are dependent on the site and dose of fractionated radiotherapy, as well as the age of the recipient, with increasing adverse effects as women age. Pelvic radiotherapy is associated with the most significant risk of future infertility (Chemaitilly *et al.*, 2006; Brämswig *et al.*, 2015) as well as increased risk of pregnancy complications (Signorello *et al.*, 2010; Van Der Kooi *et al.*, 2018). Identification of those at high risk of future gonadal failure is essential to ensure that consideration is given to fertility preservation. National and international guidelines recommend that young patients who may benefit from fertility preservation should undergo pre-treatment assessment by fertility services (Scottish Intercollegiate Guidelines Network 2013; Oktay *et al.*, 2018; Anderson *et al.*, 2020; Lambertini *et al.*, 2020).

Fertility preservation options for young females includes controlled ovarian stimulation with subsequent cryopreservation of oocytes or embryos; strategies to protect the ovaries from damage including ovarian transposition or downregulation with gonadotrophin-releasing hormone agonists; and ovarian biopsy or oophorectomy for cryopreservation (Anderson *et al.*, 2020). Ovarian tissue cryopreservation (OTC) is the only option for prepubertal girls. It involves surgical retrieval of tissue via oophorectomy or multiple ovarian cortical biopsies, and cryopreservation of this tissue. Future autologous transplantation of the tissue aims to restore fertility with the potential for natural conception, as well as restoration of hormonal function. Since the first birth after transplantation of cryopreserved ovarian cortex in 2004 (Dolmans *et al.*, 2004), there have been over 130 livebirths reported worldwide (Lotz *et al.*, 2019; Dolmans *et al.*, 2021). However, to date there have only been three live births and one ongoing pregnancy reported following re-implantation of ovarian tissue cryopreserved from three peripubertal girls; two of these patients had non-malignant diagnoses and one had a diagnosis of acute lymphoblastic leukaemia (Demeestere *et al.*, 2015; Matthews *et al.*, 2018; Rodriguez-Wallberg *et al.*, 2021). Therefore, the use of OTC and future transplantation in prepubertal girls continues to be considered experimental, requiring long-term follow-up to assess efficacy.

Correct patient selection for OTC can be challenging to avoid unnecessary surgical intervention in potentially unwell children, while not missing the opportunity for preserving ovarian tissue in those at risk. The development of a set of criteria, sometimes termed The Edinburgh selection criteria (Wallace *et al.*, 2005, 2014) (Table I), was developed to ensure that OTC is offered selectively and appropriately to those at high risk of future premature ovarian insufficiency (POI) and loss of fertility. Importantly, these criteria were developed at a time when fertility preservation in girls and young women was in its infancy and thus need to be reviewed and potentially revised in the light of emerging evidence. A previous publication reported reproductive outcomes following OTC over a 15-year period in young girls and women diagnosed with cancer (Wallace *et al.*, 2014); this indicated that the Edinburgh selection criteria remained a robust tool for identifying patients at risk of POI as a result of their cancer and its treatment, who would benefit most from OTC.

**Table I:** Edinburgh selection criteria for cryopreservation of gonadal tissue.

Edinburgh selection criteria
Age younger than 35 years No previous chemotherapy or radiotherapy if aged 15 years or older at diagnosis, but mild, non-gonadotoxic chemotherapy acceptable if younger than 15 years A realistic chance of surviving for 5 years A high risk of premature ovarian insufficiency (>50%) Informed consent (from parents and, where possible, patient) Negative serology results for HIV, syphilis, and hepatitis B Not pregnant and no existing children

Adapted from Wallace *et al.* (2005).

However, given the relatively young age of the patient cohort, ongoing follow-up of patients throughout their reproductive lifespan is required. This allows time for any effects of treatment on fertility and reproductive function to manifest, as well as taking into consideration the likely interval between cancer treatment in childhood and the desire to become pregnant. In addition, continued assessment of the suitability of these eligibility criteria, in the context of evolving treatment protocols and increasing knowledge about the impact of treatment on fertility, is required.

This is a follow-up study of this original cohort, assessing outcomes after a longer period of follow-up, with the addition of a cohort of adolescent and young girls diagnosed since the last analysis. The aim is to assess the ongoing validity of the Edinburgh selection criteria by detecting the prevalence of POI in those who were either offered or not offered OTC in a regionally defined population of patients, giving a clear denominator.

## Materials and methods

### Patient selection and study design

This study cohort consists of all female patients diagnosed with cancer before the age of 18, between 1 January 1996 and 30 April 2020 at the Edinburgh Children’s Cancer Centre, which provides care to all children diagnosed with cancer in south east Scotland. This encompasses the cohort described by Wallace *et al.* (2014), with additional patients diagnosed since then. Patients were identified from the local cancer registry. The aim of this study is to compare incidence of POI at the time of most recent assessment, in patients who were offered OTC compared to those who were not offered OTC.

At the time of diagnosis, fertility risk assessment was made and OTC offered to those patients who met the Edinburgh Selection Criteria. These criteria were established during multidisciplinary discussions on the basis of the scientific evidence then available with subsequent minor amendments (Wallace *et al.*, 2005; Anderson *et al.*, 2008). Fertility risk assessments entailed use of clinical expertise and scientific evidence to determine whether individual patients had more than a 50% likelihood of developing POI based on the intended treatment plan. When OTC was offered and accepted, written informed consent was obtained from parents/carers, and where appropriate from patients.

This study was reviewed and approved by the Lothian Research Ethics Committee (Ref 06/S1103/26).

### Data collection

A retrospective review of electronic case notes was undertaken for all eligible patients. Patients were excluded from data collection if they were aged under 12 on date of data censoring (22 October 2020) or if they died before the age of 12. These patients were excluded on the basis that they were likely to be prepubertal and therefore not suitable for clear assessment of current reproductive status.

Data about current reproductive status were gathered for all patients aged 12 years old or over on 22 October 2020. This included information about menstruation, puberty, pregnancies and reproductive hormone measurements. Presence or absence of POI was defined by documented diagnosis in the patient’s medical record, based on accepted criteria (Webber *et al.*, 2016), i.e. amenorrhoea for at least 4 months; at least two FSH measurements >25 IU/l; and/or low serum oestradiol (<150 pmol/l) in the presence of elevated FSH (>25 IU/l). Patients were assumed not to have POI if they were progressing through puberty, having regular menstrual cycles, and/or had normal serum levels of oestradiol and gonadotrophins. Pregnancy was only used to exclude POI in the absence of documented diagnosis, such that any patients who conceived prior to diagnosis of POI or who used ART due to POI would not be incorrectly categorized. Use of hormonal contraception was noted, and these patients were excluded from analysis unless it was documented that this was for hormone replacement for POI or for a diagnosis of panyhypopituitarism, in the absence of gonadotoxic treatments.

Original diagnoses were coded into the following categories: leukaemia, lymphoma, CNS solid tumour, non-CNS solid tumour, and other. Information about relapses was documented, and treatment details were recorded, where available.

### Analysis

The Kaplan–Meier method was used to carry out a time-to-event analysis, with occurrence of POI as the defined event. Patients were censored from analysis at the point of developing POI, date of last assessment, or date of death. Life tables were calculated for patients offered OTC and those not offered OTC. Patients for whom reproductive outcomes were not available were assumed not to have POI; analysis was also conducted excluding these patients, to assess whether their inclusion biased results. Hazard ratios (HRs) were calculated using Cox proportional hazards method. Subgroups were compared using Welch’s *t*-test. Statistical analysis was conducted using the tidyverse, finalfit, and survival packages for R version 4.0.5 in RStudio version 1.4.1103 (Therneau and Grambsch, 2000; Wickham *et al.*, 2019; Harrison *et al.*, 2020; R Core Team, 2021; Therneau, 2021).

## Results

### Patient cohort

**Figure 1.**
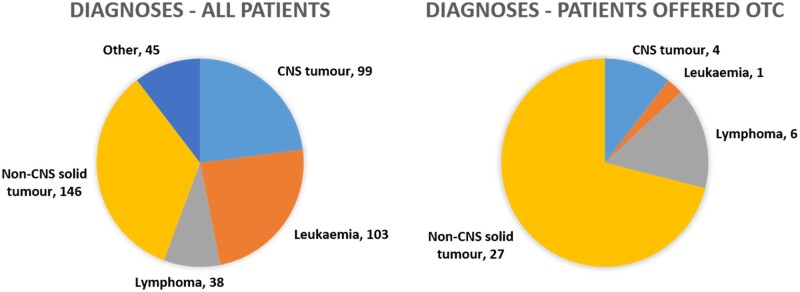
**Breakdown of diagnoses in all patients and in patients offered ovarian tissue cryopreservation.** CNS: central nervous system; OTC: ovarian tissue cryopreservation.

A total of 638 female patients diagnosed under the age of 18, between 1 January 1996 and 30 April 2020 were identified; this is an increase from the cohort of 410 patients (of whom 161 were eligible for analysis) we previously reported (Wallace *et al.*, 2014). Patients who died before the age of 12 (n = 73) or were under 12 at the time of data censoring (22 October 2020; n = 134) were excluded. Data were collected on the remaining 431 patients. Within this population, the median age at diagnosis was 9.8 years (interquartile range [IQR] 4.1–12.8) and median age at time of analysis was 22.2 years (IQR 16.9–27.7). The most common diagnoses were non-CNS solid tumours (n = 146, 33.9%), followed by leukaemias (n = 103, 23.9%) (Fig. 1).

Within this cohort, 38 (8.8%) patients were offered OTC and 393 (91.2%) were not offered OTC. In patients offered OTC, the most common diagnostic group was non-CNS solid tumours (n = 27) (Fig. 1). While there have been evolutions in treatment protocols and our understanding of the gonadotoxicity of different treatment regimens over time, analysis of diagnoses over time showed that patients with non-CNS solid tumours are consistently the group to whom OTC was most frequently offered. While 37 patients were offered OTC at the point of initial diagnosis, one patient was offered OTC at relapse.

Of those offered OTC, 26 accepted and 12 declined; the procedure was unsuccessful in one patient; therefore a total of 25 patients successfully underwent OTC at a median age of 11.7 years (IQR 9.6–13.8). The procedure was carried out laparoscopically in all cases, in combination with another surgical procedure whenever possible. There were no surgical complications.

Detailed information about treatments received was available for 105 patients. Of these, 18 (17%) patients received high dose alkylating chemotherapy (defined as cyclophosphamide equivalent dose >6000 mg/m^2^; Mulder *et al.*, 2021), one received total body irradiation, and two received pelvic irradiation. Thus, a total of 20% of these patients received these high-risk treatments.

### Reproductive outcomes

The median duration of follow-up for the total cohort was 7.2 years (IQR 2.5–12.2). For the subgroup of patients offered OTC, the median duration of follow-up was 8.1 years (IQR 3–12.4), and for those not offered OTC, it was 7.0 years (IQR 2.4–12.2).

**Figure 2.**
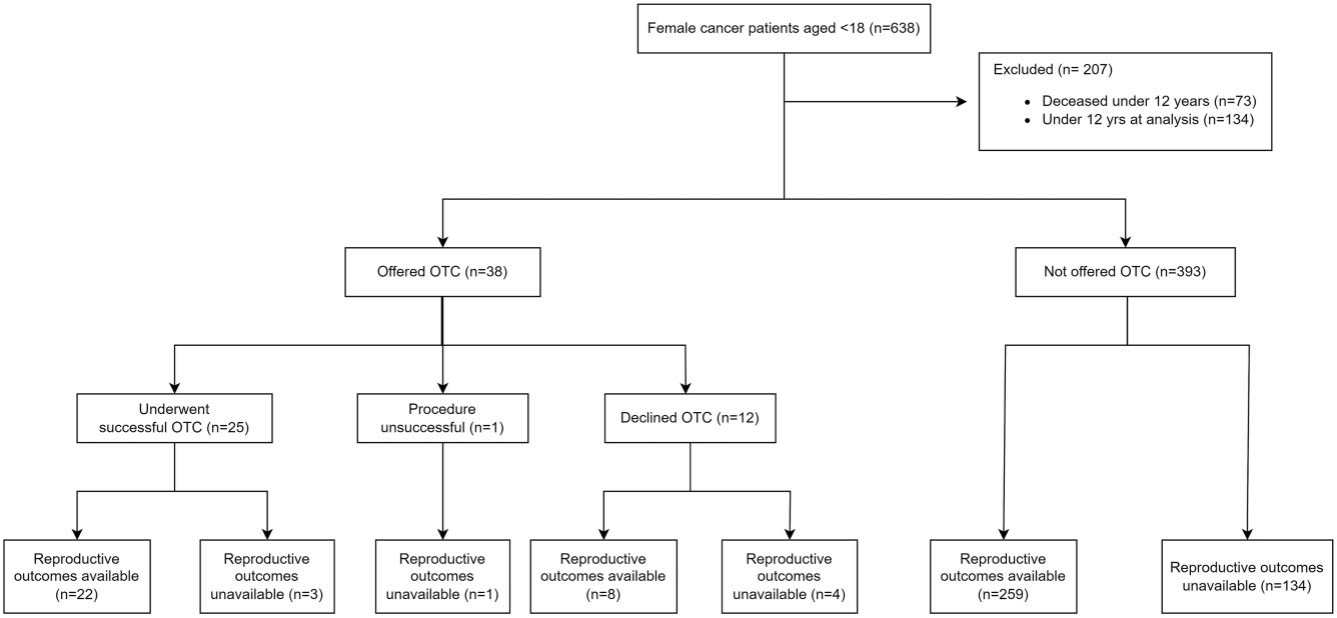
**Participant profile.** OTC: ovarian tissue cryopreservation.

Information about current reproductive status outcomes was available for 289 (67.0%) and unavailable for 142 patients (Fig. 2). Of these 142 patients, 42 (29.6%) have had clinical contact in the 5 years preceding the study date, but no reproductive outcomes were documented. Eight of the 142 patients had been offered OTC. Two of these patients are now deceased; one has moved out of the area; four have documented clinical contact in the last 5 years; and one is lost to follow-up. Three of the eight patients successfully underwent OTC, and these patients remain under follow-up but have not had reproductive outcomes documented.

We tested the assumption that in the absence of documented POI, these 142 patients could be classified as not having POI. To assess for risk of bias, the two groups (those with known reproductive outcomes and those with unknown reproductive outcomes) have been compared for baseline characteristics (Table II). This showed that age at diagnosis, age at analysis, and interval since diagnosis were similar in those with and without known reproductive outcome, whereas duration of follow-up was much shorter in those with unknown reproductive outcome. Given these similarities, analyses were performed excluding this group, with a second analysis assuming that the group with unknown outcome did not have POI.

**Table II:** Comparison of patients with known and unknown current reproductive outcomes.

	Reproductive outcomes known	Reproductive outcomes unknown	*P* value
n	289	142	
Age at diagnosis	9.8 (4.5–13.6)	9.3 (2.85–11.9)	0.049
Age at analysis	22.5 (17.4–27.6)	21.7 (16.2–27.8)	0.640
Years since diagnosis	13.0 (8.2–18.9)	15.3 (8.9–19.1)	0.281
Duration of follow-up	10.1 (5.2–13.7)	2.0 (0.2–5.5)	<0.0001

Values are represented as median (IQR). “Years since diagnosis” is defined as the number of years from date of diagnosis to date of analysis. “Duration of follow-up” is defined as the number of years from date of diagnosis to date of last documented clinical follow-up. Data are compared using Welch’s *t*-test.

Information about reproductive outcomes is shown in Table III. When multiple outcomes were available, the most recently documented outcome was recorded. There have been a total of 75 pregnancies across 42 patients. Maternity records were available for 25 (59.5%) of these patients and confirm that donor eggs were not used; the remaining 17 patients had their maternity care out of area. Four pregnancies have occurred in patients who were offered OTC (none using stored tissue) and three in patients with a confirmed diagnosis of POI. Thirteen patients had a documented diagnosis of panyhypopituitarism; all of these patients were treated for brain tumours without gonadotoxic treatment and none had a documented diagnosis of POI. Therefore, all were considered not to have POI. Nine patients were noted to be taking hormonal contraception without documentation of POI and were excluded from ongoing analysis; this includes one patient who was offered OTC. Twenty patients have a diagnosis of POI (4.6%).

**Table III:** Reproductive outcomes of all patients aged >12 at study date, including those deceased aged >12.

Reproductive outcome	Number of patients (%) (n = 431)
Premature ovarian insufficiency*	20 (4.6%)
Basis for classification as ‘not POI’	
Menstruation recorded	166 (38.5%)
Pregnancies** recorded	31 (7.2%)
Hormonal measurements	29 (6.7%)
Evidence of pubertal development (pre-menarche)	21 (4.9%)
Panhypopituitarism	13 (3.0%)
Hormonal contraception	9 (2.1%)
No reproductive outcomes available	142 (32.9%)

* Patients were classified as having premature ovarian insufficiency (POI) if this diagnosis was documented in the electronic medical record and based on accepted guidelines (Webber *et al.*, 2016). In patients without a documented diagnosis of POI for whom multiple reproductive outcomes were available, the most recent outcome has been used.

** Pregnancy was only used to exclude POI in the absence of a documented diagnosis of POI.

### Patients offered OTC were more likely to develop POI

In the 422 patients aged >12 and not taking hormonal contraception, OTC was offered to 37 patients, with a median age at time of diagnosis of 11 years (IQR 7.8–13.6). The most common diagnosis in this group of patients was Ewing’s sarcoma (n = 10), followed by Hodgkin’s lymphoma (n = 6) and rhabdomyosarcoma (n = 6). Current reproductive outcomes of patients offered OTC are summarized in Table IV.

**Table IV:** Reproductive outcomes of patients offered ovarian tissue cryopreservation.

Reproductive outcome	Number of patients *(%)* (n = 38)
Current menstruation recorded	12 *(5%)*
Pregnancies recorded	2 *(%)*
Hormonal measurements	5 *(%)*
Evidence of pubertal development (pre-menarche)	1 *(%)*
Premature ovarian insufficiency	9 *(%)*
Panhypopituitarism	0 *(0%)*
Hormonal contraception	1 *(%)*
No reproductive outcomes available	8 *(%)*

**Figure 3.**
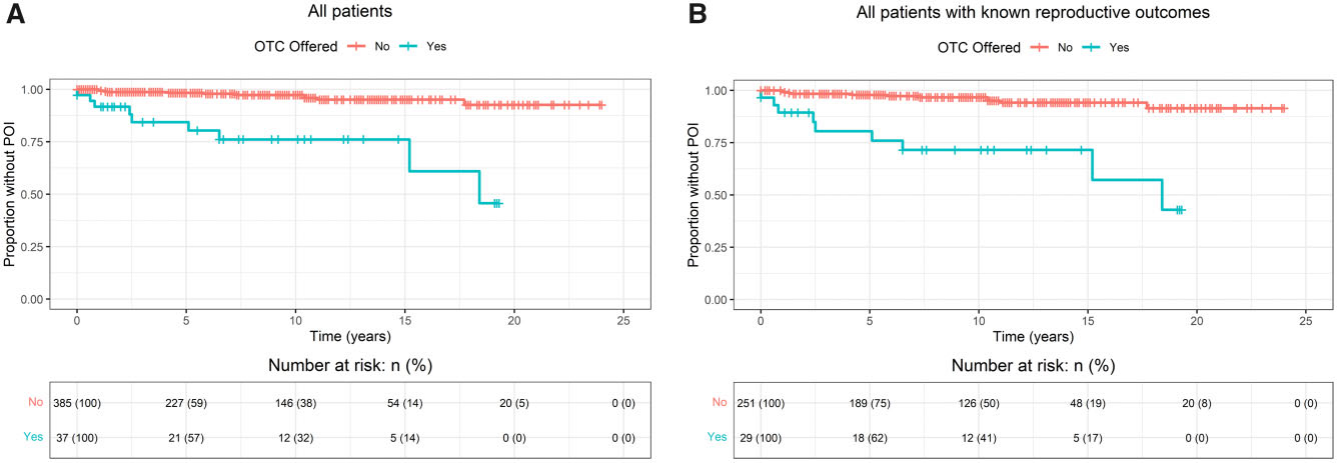
**Cumulative probability of not developing POI in all patients (A) and only in patients for whom reproductive outcomes are known (B).** Data compared using Kaplan–Meier method and hazard ratios generated using Cox proportional hazards model. OTC: ovarian tissue cryopreservation; POI: premature ovarian insufficiency.

In the group of patients who were offered OTC, there were nine cases of POI (24.3%). In the 385 patients not offered OTC, there were 11 cases of POI (2.9%). The median time from diagnosis to developing POI was 4.7 years (IQR 1.2–10.4). The cumulative probability of developing POI was thus significantly higher in the group of patients offered OTC than in those not offered OTC (HR 8.7 [95% CI 3.6–21]; *P* < 0.0001; Fig. 3A). When patients with unknown reproductive outcomes were excluded, the cumulative probability of developing POI remained significantly higher in the cohort offered OTC, with an incidence of 31.0% compared to 4.4% (HR 8.1 [95% CI 3.4–20]; *P* < 0.001; Fig. 3B). Schoenfeld residuals were plotted to test proportional hazards assumption (Supplementary Fig. S1).

In the group offered OTC (n = 37), all patients who developed POI did so after treatment for primary disease; whereas in the 11 patients who had not been offered OTC and developed POI, five patients developed POI after treatment for disease relapse. These patients who relapsed had diagnoses of leukaemia (n = 3), medulloblastoma (n = 1), and neuroblastoma (n = 1). Detailed treatment information was available for eight patients with POI; of these, seven (87.5%) had high dose alkylating treatment.

## Discussion

OTC is an important fertility preservation option for females. Indeed, in 2019 the American Society of Reproductive Medicine (ASRM) published a committee opinion statement on fertility preservation in patients undergoing gonadotoxic therapy, recommending that OTC is an established medical procedure and should no longer be considered experimental (Practice Committee of the American Society for Reproductive Medicine, 2019). However, the 2020 ESHRE guideline recommended it be regarded as ‘innovative’, due to the limited data on safety and efficacy (Anderson *et al.*, 2020), and it is important to acknowledge the paucity of data about outcomes in prepubertal and adolescent patients. Our data show that in this population of female patients diagnosed with childhood cancer under the age of 18 years, the overall prevalence of POI is low, at 4.7%; this is in line with the risk of POI following childhood cancer in previous studies (Thomas-Teinturier *et al.*, 2013; Levine *et al.*, 2018). Given the overall positive fertility outcomes in these patients (Chow *et al.*, 2016) and the limited data available on outcomes after re-implantation of cryopreserved tissue in this patient group, it is important that OTC is offered only after individualized assessment.

The Edinburgh selection criteria offer a tool to stratify risk and identify those that would benefit most from this procedure. These criteria were introduced in 1996 following extensive multidisciplinary discussion. At that time, OTC was a novel procedure and it was anticipated that the criteria would change over time, as knowledge and treatments for cancer evolved. The criteria underwent minor revision in 2000 but have largely remained the same since then. There are limited data available on female reproductive function following childhood cancer treatment to inform such revisions, and here we present a set of outcomes over a 24-year period, with an unbiased population based denominator. The results of this review demonstrate an increased lifetime risk of POI in the cohort offered OTC, with an overall prevalence of 24.3% compared to 2.9% in those not offered OTC. This confirms that the criteria remain an accurate assessment tool to support selective use of OTC as a fertility preservation option.

We are aware of three amended criterion sets published since 2018. The Chinese Society of Gynecological Endocrinology (Ruan, 2018) recommend that OTC should take place at least 3 days prior to chemotherapy or radiotherapy, while the Oxford, UK service do not specifically exclude on the basis of having had previous gonadotoxic chemoradiotherapy or having existing children (Lakhoo *et al.*, 2019). While the Edinburgh criteria do exclude patients who have existing children, it is important to note that this is not related to the safety or efficacy of the procedure, but rather is in line with national access criteria for ART. Most recently, a Japanese publication presented revised criteria with additional focus on ovarian metastasis/invasion and the requirement of an intact uterus, in order to align with Japanese laws prohibiting surrogacy (Takae *et al.*, 2022).

Given the evolving nature of cancer treatments and our understanding of their effects on fertility, it follows that any eligibility criteria for fertility preservation in this patient cohort must be continually re-assessed. This analysis highlights two areas for consideration: the need for ongoing patient follow-up and the challenge of disease relapse.

With a median age at diagnosis of 9.8 years and at analysis of 22.2 years, this remains a relatively young population. It is anticipated that the rates of POI in this group will rise with increasing time from diagnosis and treatment, and patients who have progressed through puberty and not yet considered starting a family may have no indication of the effects of their treatment on their ovarian function. A study has shown that 77% of survivors of childhood cancer were unaware of their current fertility status (Lehmann *et al.*, 2017), demonstrating low levels of fertility-related knowledge. While the current rate of POI in the cohort offered OTC is lower than the risk suggested by the Edinburgh selection criteria, this is likely reflective of the challenges of predicting risk accurately and the still relatively young age of the cohort and emphasizes the need for ongoing assessment of this population. In addition, although the rates of POI in the population not offered OTC are relatively low, this group does represent a population who have gone on to develop a treatment complication that was initially considered to be unlikely or low risk. Accurate estimation of risk is challenging, which is why patient counselling about possible fertility outcomes, appropriate clinical follow-up, and long-term follow-up studies of this patient cohort (including assessment of remaining ovarian function as well as POI) are essential.

Of note, reproductive outcomes were unavailable in 32.9% of this cohort. Many of these patients continue to be under haematology/oncology follow-up, with 42 of the 143 (29.4%) having had a hospital appointment in the last 5 years, while others have moved out of area or been discharged from routine follow-up. It could be assumed that patients under regular follow-up have normal reproductive function, which has simply not been documented; however, this highlights the need for routine assessment and documentation of reproductive function to be part of standardized cancer after care (Anderson *et al.*, 2021). Other studies have acknowledged the potentially problematic nature of follow-up, either due to unknown contact details or an unwillingness to participate in patient questionnaires (Jadoul *et al.*, 2017). By reviewing electronic records, we overcame the issue of patient uptake of a survey and ensured that the whole population was assessed, avoiding the bias of patient selection by recruitment.

Another important challenge highlighted by this review is that of disease relapse. All the patients who developed POI in the group that was offered OTC did so after treatment for the primary disease, highlighting that accurate identification of risk had taken place in those patients. In comparison, almost half (45.5%) of the patients with POI in the group not offered OTC developed this after treatment for disease relapse. The Edinburgh Selection Criteria specify that patients under the age of 15 who have had non-gonadotoxic chemotherapy should still be considered eligible for OTC (Wallace *et al.*, 2005). However, patients presenting with relapsed disease may be acutely unwell and therefore medically unfit for OTC, or the urgency of starting secondary treatment may exclude this as an option. The most common diagnosis in this group was leukaemia; a further complication with this group at the time of both initial diagnosis and relapse is the risk of ovarian tissue contamination with malignant cells (Rosendahl *et al.*, 2013; Dolmans and Masciangelo, 2018), precluding subsequent autotransplantation, although there are case reports of successful treatment where ovarian tissue was collected during remission (Shapira *et al.*, 2018). A further discussion of fertility preservation options should occur taking into account the individual patient’s health status, the gonadotoxicity of previously received treatment and personal wishes.

The use of OTC for fertility preservation is increasing worldwide with variable indications for its usage. The lack of an international register means that the number of OTC procedures performed worldwide is unknown. This unknown denominator makes the efficacy of the procedure hard to assess. Most studies quote estimated pregnancy rates of around 30% after future autograft of tissue (Jadoul *et al.*, 2017; Dolmans *et al.*, 2021). However, data almost exclusively relate to outcomes in post-pubertal patients, with only three published case reports of livebirth after autograft of tissue cryopreserved during childhood (Demeestere *et al.*, 2015; Matthews *et al.*, 2018; Rodriguez-Wallberg *et al.*, 2021). Two of these patients had non-malignant indications for OTC (sickle cell anaemia and beta thalassaemia) and one had a diagnosis of leukaemia. Outcomes from ovarian tissue cryopreserved post-puberty cannot be directly extrapolated to prepubertal patients, given that immature ovarian tissue has been demonstrated to contain a higher proportion of abnormal follicles and oocytes from young women have higher rates of aneuploidy (Anderson *et al.*, 2014; Gruhn *et al.*, 2019). Therefore, there is clear need for ongoing research into this patient population to assess their specific outcomes, utilization of stored tissue, and efficacy of transplantation.

A large study from Brussels reported that 3.9% of their population went on to utilize stored tissue, while 12% requested the tissue be donated or destroyed. However, the follow-up time was only 7.6 years and OTC was not limited to only patients at high risk of POI. The authors acknowledge that within their cohort there was a group in whom the procedure was ‘currently of little value’ as they had subsequently died, had persistent ovarian function, or had conceived without use of their stored tissue (Jadoul *et al.*, 2017). Within our patient cohort, only one patient has thus far requested re-implantation of stored tissue (with evidence of restoration of ovulation but no pregnancy at present); however, given the relatively young median age of the population offered OTC (23.1 years) it is likely that further patients will go on to request auto-transplantation.

For some patients, future autologous transplantation of their tissue is not currently recommended. This is usually in the context of leukaemia and concerns about the potential to re-seed malignant cells. For this group, IVM of oocytes may be a future viable option. Immature oocytes from antral follicles in the ovarian tissue can be matured in vitro and cryopreserved as mature oocytes. Oocyte aspiration for IVM is now a recognized technique, and recovering immature oocytes from ex-vivo of ovarian tissue is possible, and sometimes referred to as ovarian tissue oocyte IVM (OTO-IVM). A study of 77 patients undergoing OTO-IVM, including nine prepubertal girls, demonstrated an oocyte maturation rate of 42% for adult patients and 22% in prepubertal patients; there have been three live births after OTO-IVM in this cohort, all from patients whose tissue was obtained post-pubertally (Segers *et al.*, 2020). IVM will hopefully expand the future reproductive options of this cohort of young girls and women; however, this procedure remains experimental and outcomes in prepubertal girls remain uncertain.

From the patient perspective, future fertility has been shown to be a key concern of survivors of childhood cancer, with a strong desire to have their own biological children (Lehmann *et al.*, 2017) and there are high levels of satisfaction reported following OTC, even when tissue has not been utilized (Jadoul *et al.*, 2017). The data presented here support the continued selective use of OTC as a fertility preservation method for young girls and adolescents diagnosed with cancer, with identification of those at increased risk of POI compared to the low overall prevalence of under 5%. In prepubertal girls, OTC should continue to be considered experimental until more robust evidence is available assessing outcomes after future autografting of the tissue. An international register would help to determine the overall efficacy of OTC, particularly in these very young patients, potentially enhancing patient selection and counselling; ESHRE are now collecting these data through the European IVF monitoring (EIM) programme. It is necessary to continue to follow up our cohort to determine longer-term reproductive outcomes as well as pregnancy outcomes after future autografting of tissue.

## Supplementary Material

dead060_Supplementary_Figure_S1

## Data Availability

The data underlying this article cannot be shared publicly for the privacy of the individuals that participated in the study. The data will be shared on reasonable request to the corresponding author.
