# Supplementary material for: Long-term follow-up to assess criteria for ovarian tissue cryopreservation for fertility preservation in young women and girls with cancer
Source: Hum Reprod. 2023 Apr 3;38(6):1076–85. doi: 10.1093/humrep/dead060 (PMC10233253; doi:10.1093/humrep/dead060)
Supplement: dead060_Supplementary_Figure_S1 [file dead060_supplementary_figure_s1.pdf]

Human Reproduction, pp. 1–1, 2023
https://doi.org/10.1093/humrep/dead060

human reproduction

SUPPLEMENTARY DATA

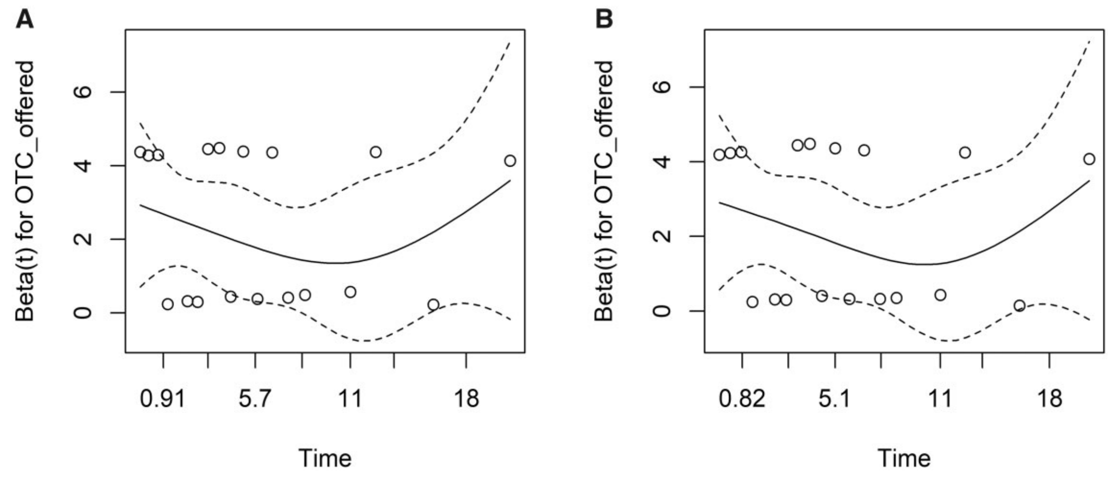

Supplementary Figure S1. Schoenfeld residuals plots for Cox proportional hazards calculation used to generate hazard ratios for all patients (A) and only in patients for whom reproductive outcomes are known (B). An assumption of the Cox proportional hazards model is that hazards are constant over time; using these Schoenfeld residuals against the transformed time, we demonstrate that this proportionality assumption is being met. OTC: ovarian tissue cryopreservation.
